# Reverse Taxonomy for Elucidating Diversity of Insect-Associated Nematodes: A Case Study with Termites

**DOI:** 10.1371/journal.pone.0043865

**Published:** 2012-08-28

**Authors:** Natsumi Kanzaki, Robin M. Giblin-Davis, Rudolf H. Scheffrahn, Hisatomo Taki, Alejandro Esquivel, Kerrie A. Davies, E. Allen Herre

**Affiliations:** 1 Fort Lauderdale Research and Education Center, University of Florida/IFAS, Davie, Florida, United States of America; 2 Forestry and Forest Products Research Institute, Tsukuba, Ibaraki, Japan; 3 Universidad Nacional, Escuela de Ciencias Agrarias, Laboratorio de Nematologia, Heredia, Costa Rica; 4 Centre for Evolutionary Biology and Biodiversity, School of Agriculture, Food and Wine, The University of Adelaide, Waite Campus, Glen Osmond, South Australia, Australia; 5 Smithsonian Tropical Research Institute, Balboa, Ancon, Republic of Panama; George Washington University, United States of America

## Abstract

**Background:**

The molecular operational taxonomic unit (MOTU) has recently been applied to microbial and microscopic animal biodiversity surveys. However, in many cases, some of the MOTUs cannot be definitively tied to any of the taxonomic groups in current databases. To surmount these limitations, the concept of “reverse taxonomy” has been proposed, i.e. to primarily list the MOTUs with morphological information, and then identify and/or describe them at genus/species level using subsamples or by re-isolating the target organisms. Nevertheless, the application of “reverse taxonomy” has not been sufficiently evaluated. Therefore, the practical applicability of “reverse taxonomy” is tested using termite-associated nematodes as a model system for phoretic/parasitic organisms which have high habitat specificity and a potential handle (their termite host species) for re-isolation attempts.

**Methodology:**

Forty-eight species (from 298 colonies) of termites collected from the American tropics and subtropics were examined for their nematode associates using the reverse taxonomy method and culturing attempts (morphological identification and further sequencing efforts). The survey yielded 51 sequence types ( =  MOTUs) belonging to 19 tentatively identified genera. Within these, four were identified based on molecular data with preliminary morphological observation, and an additional seven were identified or characterized from successful culturing, leaving eight genera unidentified.

**Conclusions:**

That 1/3 of the genera were not successfully identified suggests deficiencies in the depth of available sequences in the database and biological characters, i.e. usually isolated as phoretic/parasitic stages which are not available for morphological identification, and too many undiscovered lineages of nematodes. Although there still is the issue of culturability of nematodes, culturing attempts could help to make reverse taxonomy methods more effective. However, expansion of the database, i.e., production of more DNA barcodes tied to biological information by finding and characterizing additional new and known lineages, is necessary for analyzing functional diversity.

## Introduction

Molecular sequence-based approaches have altered how scientists are approaching biodiversity surveys of micro- and meiofauna [1]–[6]. Traditionally, surveys of regional or geographic diversity were based on collection, observation and identification of the macrofauna by specialists, i.e. insect traps by entomologists, line/aerial census for vertebrates, plants and mushrooms by ornithologists, mammalogists, botanists and mycologists, with microbes and microscopic metazoans being largely ignored because of the excessive amount of time required for identification [7]. The molecular operational taxonomic unit (MOTU)-based survey, where every kind of organism is recognized as a taxon-specific molecular sequence, does not require any special knowledge and skill to distinguish a particular group of organisms. Further, some of the MOTUs can be putatively tied to a taxonomic rank or “species” or “genus” ( =  scientific name) quickly and accurately using sequence databases, e.g. GenBank, if these sequences are available in the database and accurately identified therein ( =  DNA barcodes). Recent environmental DNA and pyrosequencing techniques are increasingly being evaluated for large-scaled surveys of microbes and microscopic animals [8]–[14]. The surveys of these small and divergent organisms would have been almost impossible with traditional methods, i.e. isolation and identification/description for each species from the field.

MOTU-based analysis is not without problems for surveys of microbes and microscopic metazoans. For example, the available sequence length for MOTU analysis is usually ca 400 (pyrosequencing) [11], [12] to at most <2000 (environmental DNA sequencing) [4] base pairs, which is sometimes suboptimal for precise phylogenetic positioning of each MOTU, and the reference sequences of these organisms are often not available in sufficient breadth and depth or worse yet, if present, are misidentified [5], [6], [11], [15]. Further, if the DNA barcodes were randomly sequenced from environmental DNA, there are no voucher specimens available for confirmation of its taxonomic status. Thus, in many cases, some MOTUs cannot be definitively tied to any of the taxonomic groups, and those MOTUs should be treated as “unknown MOTUs” classified into “unknown clades”. Therefore, even if the lists of MOTUs and their closest hit in GenBank are generated in a study, the composition of trophic groups and potential interactions among these MOTUs would be poorly estimated.

To complement the taxonomic and ecological information in diversity surveys, the concept of “reverse taxonomy” has been proposed, i.e. to primarily list the MOTUs with photo-documentation, and then use subsamples obtained together with MOTU materials, or re-isolate the organisms from specific substrate(s) or host(s) according to the MOTU analysis information to identify and/or describe them at genus/species level [16]–[18]. By this operation, presence/absence and number of new or unknown lineages and their specific habitat and/or host can be hypothesized. Subsequently, the new or unknown lineage may be re-isolated during an intensive survey on the target substrate and/or hosts to elucidate their functional roles and interactions in the ecosystem.

Currently, although some successful cases have been reported [19], the practical application of “reverse taxonomy” has not been sufficiently understood.

In the present study, to test the practical applicability of “reverse taxonomy” to the potentially large number of insect-associated nematodes (>200,000 species), we examined the diversity of termite-associated nematodes in the North American meridian as a model system using MOTU-based and isolation (culturing)-based analyses.

Powers et al. [6] examined nematode biodiversity in soil, epiphytes, plants and insects in La Selva, Costa Rica. In the La Selva survey, termites (Isoptera) were chosen as a focal sampling group for entomophilic nematodes. Seven nematode MOTUs in total were identified from dissections of termites from a transect, with none of them overlapping those identified from soil, epiphytes and plants from the same transect. This suggested that termite-associated nematodes were intricately bound to the heterogeneous microniches of their hosts and that their hosts therefore represented a sampling or “reverse taxonomy” handle for a more predictable way to revisit and re-isolate the nematodes to build a body of information to associate with and strengthen the MOTU. We therefore propose that termite-associated nematodes are a good model system to test the applicability of “reverse taxonomy” for host-associated nematodes.

## Results

Forty seven species (259 colonies) and 15 species (39 colonies) of termites were collected and dissected during the MOTU/preliminary morphological observation and culturing surveys, respectively (Tables S1–S3).

During the dissection and direct isolation of nematodes, except for four morphospecies of thelastomatid parasites, most of the nematodes were associated with the insects as the dauer (phoretic) or parasitic juvenile stages and were not morphologically identifiable. From the 259 individual colonies of termites, 159 individual nematodes were picked up and processed into digestion, PCR amplification and sequencing. One hundred thirty individual nematodes out of 159 were successfully sequenced and separated into 42 MOTUs, four morphospecies of thelastomatid parasites and an unidentified diplogastrid species, which were not sequenced successfully. Within these MOTUs, *Poikilolaimus floridensis* and *Rhabditis rainai* were each established as a laboratory culture and described and identified, respectively, based on morphology and near full length SSU ribosomal DNA sequencing [20], [21].

**Figure 1:**
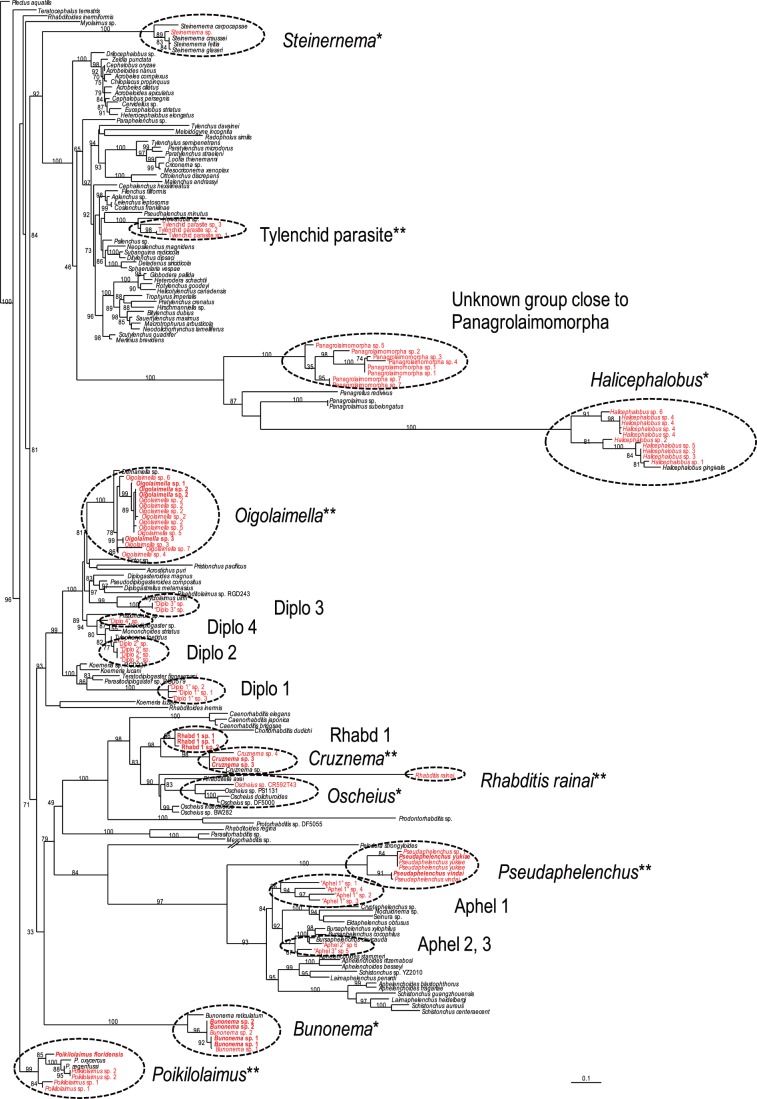
Molecular phylogenetic relationship among MOTUs and the SSU sequences stored in the GenBank database. The 100001st Bayesian tree inferred from MOTUs and SSU sequences under GTR+I+G model (lnL = 30163.4492; freqA = 0.2367; freqC = 0.2089; freqG = 0.2585; freqT = 0.2959; R(a) = 1.1766; R(b) = 2.7362; R(c) = 1.8858; R(d) = 0.6747; R(e) = 4.2046; R(f) = 1; Pinva = 0.1854; Shape = 0.57). Posterior probability values exceeding 50% are given on appropriate clades. Successfully cultured species are written in bold. *: Identified solely by molecular sequence; ** : identified based on morphological observation.

**Figure 2:**
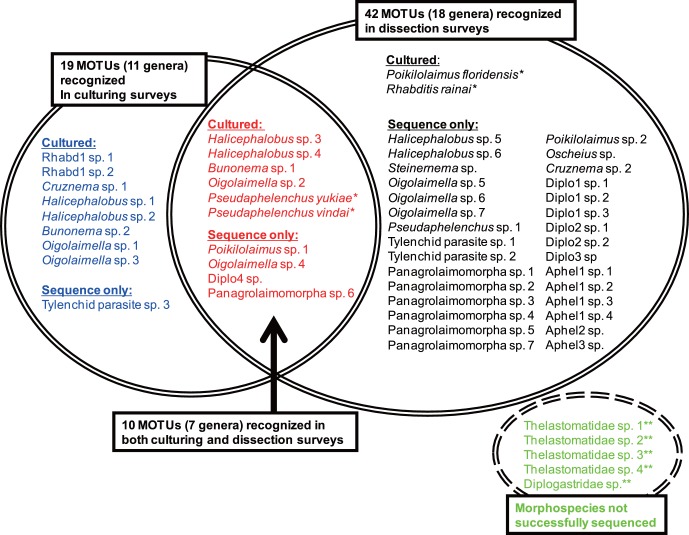
Comparison of methodologies for isolation of MOTUs. Fifty one MOTUs and five morphospecies were recognized during the surveys. Within these MOTUs, 19 and 42 were found in culturing (left circle; blue and red) and dissection (right circle; black and red) survey, respectively, and 10 were recognized by both types of surveys (center; red). *: Hand-picked during dissection analysis; **: Both sequencing and culturing not successful for five morphospecies (right bottom; green).

The MOTUs were classified into 18 phylogenetic groups ( =  tentative “generic” level identifications), and four of them, *Bunonema*, *Steinernema*, *Halicephalobus* and *Oscheius*, were identified solely by molecular sequences, i.e. these sequences were very close (>97% similarity) to the sequences of each corresponding genus. But the others, excluding the two cultured species, *P. floridensis* and *R. rainai*, were not clearly identified molecularly because of the shortage of reference sequences in the databases at the time (Figs.1, 2), i.e. they were regarded to be an unknown rhabditid, an unknown tylenchid insect parasite, five unknown diplogastrids, four unknown aphelenchs, and an unknown panagrolaimid.

Using the culturing approach, 19 MOTUs (nine tentative “genera”) were recognized, and 14 of them were successfully cultured (Figs. 1, 2). The 14 successfully cultured species were as follows: *Halicephalobus* sp. 1, 2, 3 and 4, *Bunonema* sp. 1 and 2, *Oigolaimella* sp. 1, 2 and 3, *Cruznema* sp. 1, unidentified rhabditid genus (“Rhabd 1” in Figs. 1 and 2) sp. 1 and 2 and *Pseudaphelenchus yukiae* and *P. vindai*. Within these 19 MOTUs, 10 of them overlapped with those obtained during the dissection survey and three of the genera (*Oigolaimella*, *Cruznema* and *Pseudaphelenchus*) were identified as nominal taxonomic groups and “Rhabd 1″, which were found only in the culturing method, was characterized as a group of bacteria feeders (Figs. 1, 2).

The 19 tentatively recognized genera were separated into free-living fungal feeder (*Pseudaphelenchus*), entomopathogen (*Steinernema*), insect parasite (Tylenchid parasite) and free-living bacteria feeders (*Poikilolaimus*, *Rhabditis*, *Oscheius*, *Halicephalobus*, *Oigolaimella*, *Bunonema*, *Cruznema* and Rhabd1) [22], [23]. However, the feeding resources for the other eight genera, Aphel1-3, Diplo1-4 and Panagrolaimomorpha were not specified or clearly delineated because of low taxonomic resolution.

## Discussion

Nematodes (and many other microscopic animals, e.g. mites) comprise myriad phoretic and parasitic species with various feeding habitats, i.e. these organisms have closely synchronized relationships with other organisms. Therefore, to estimate the “biodiversity” of these meiofauna communities requires not only a simple species (MOTU) list, but some information about the putative function (functional group) and their association patterns with hosts and other microbes [24].

In the case of nematodes, their biological characters are generally represented by the genus/species name. For example, the family Aphelenchoididae, some of which were found in the present study, contains mycophagous free-living species (e.g. *Aphelenchoides*), ecto- and endoparasites of insects (e.g. *Ektaphelenchus* and *Entaphelenchus*), insect-phoretic plant parasites (e.g. *Schistonchus*) and predators (e.g. *Seinura*) [25], [26], and another family, Diplogastridae is known to have evolved from a free-living bacteria feeder, contains insect parasites (e.g. *Parasitodiplogaster*), insect-phoretic fungal feeder/predators (e.g. *Neodiplogaster*), insect-phoretic fungal feeders (e.g. *Tylopharynx*), insect-phoretic bacteria feeders (e.g. *Pseudodiplogasteroides*), insect-phoretic bacteria feeder/predators (e.g. *Pristionchus* and *Mononchoides*) [27]–[32]. Further, their biological characters are not always correlated with their phylogenetic relationship within the family [22], [25]–[32]. Therefore, the identification at the family or higher rank does not provide sufficient information to evaluate their functional diversity.

In the present study, we applied the reverse taxonomy method to biodiversity surveys of termite-associated nematodes, and several expected and unexpected difficulties in integrated taxonomic procedures arose [33].

The primary and expected shortfalls were the lack of available and accurately identified sequences in the database and the applicability of “universal” primers. First, only four of the tentative 19 genera were identified by comparison of MOTUs with molecular barcodes stored in the database, but most of the MOTUs did not show sufficiently high similarity to any of the barcode sequences, and were identified at family, superfamily or infraorder level. This is partially because the length of the chosen MOTU sequence (ca. 600 bps) was not sufficient for estimating precise phylogenetic position of nematodes. For example, two unknown aphelenchoidid genera (*Pseudaphelenchus*, “Aphel1”, “Aphel2” and “Aphel3” in Fig.1) were rather close to the genus *Bursaphelenchus*, which is not likely to be associated with termites [34] in the database homology search. Also, during direct isolation, although four morphospecies of thelastomatid parasites were confirmed, none of them were successfully sequenced, probably because the primer set was unacceptable for amplification or sequencing this nematode group. As mentioned previously [5], [11]–[13], [35], the development of universal primer sets is not easy for nematodes because of their high sequence divergence rates. This also could be a limiting factor in sequence-based analysis, and appropriate primer sets are needed to amplify larger fragments with sufficient phylogenetic resolving power.

The morphological characters tied to the MOTU were expected to potentially overcome the shortcomings of the MOTU analysis, but this was not realized for termite-associated nematodes (and probably many other insect associated nematodes) because of the life history traits of these nematodes. In the dissection and hand-picking of nematodes, except for the four thelastomatid parasites, all of the nematodes were isolated as dauer (dispersal) or parasitic juveniles, which do not have genus/species-specific diagnostic characters, and were only identifiable at the family or higher taxonomic rank [36]. Therefore, although an insect parasitic genus, “Tylenchid parasite” was characterized as a genus (undescribed or not sequenced yet) close to *Howardula*, the others were not identified or characterized by the reverse taxonomy approach. These dispersal forms also complicate the reverse taxonomy procedures even with potential help from culturing attempts mentioned below. For example, *Coptotermes testaceus* is associated with eight genera (14 species) of nematodes (Table S2), and the culturing is assumed to start with multiple species with different numbers of individuals which may give a biased result due to swamping of the minor or K-strategist species. Similar phoretic stages are also known in other microscopic animals, e.g. many species of insect-associated mites propagate in their host insects’ habitat and are phoretically carried as dispersal stages, although these phoretic stages often have genus/species specific characters [37].

In the above case, culture-based morphological identification helped identify, one of the unknown diplogastrids as the bacterial-feeding free-living genus *Oigolaimella* [38], and one of the unknown aphelenchs as a new mycophagous free-living genus which was successfully described by the authors in previous papers as *Pseudaphelenchus* [22], [39]. These identifications enabled us to increase our understanding of their biological traits and potential ecological roles and interactions. In addition, *Cruznema* was also successfully identified by cultured specimens, and another genus, “Rhabd1” was considered to be a bacteriophagous genus close to *Choriorhabditis* based on the cultured materials.

However, regardless of these tandem approaches, eight of 19 tentative genera, especially, “Aphel1”, “Diplo1” and “Panagrolaimomorpha” which have wide host/carrier ranges, were not successfully isolated from any of the termites as pure cultures. This may suggest that these genera are fastidious and difficult to culture, e.g. parasitic/predatory species or require specific feeding resources. More careful dissections may help to clarify the species/genus status of these groups. It may also be necessary to collect and sequence adults from the nest of these termite species to link morphology to the MOTUs.

Overall, MOTU-based analysis has proven to be a useful tool for constructing an inventory of termite-associated nematodes to assess association rates and insect-associated nematode diversity. High through-put pyrosequencing analysis is also a highly effective method for dealing with small and abundant organisms, i.e. microbes and microscopic animals in environmental samples [9], [12]–[14]. However, currently, there is a lack of breadth and depth of microbe and microscopic invertebrate sequences tied to a reliable and sufficient body of biological information in the database. Thus, molecular sequence-based diversity analysis is still somewhat disconnected from the function and biology of the organisms that are being studied.

In the present and previous studies, we demonstrated that a MOTU/morphology survey (reverse taxonomy) followed by re-isolation and culturing attempts improved the efficiency of identification and led to the discovery of new species and genera to science and improved the resolution of the database for future work [21], [22], [39]. Thus, we consider that the reverse taxonomy method effectively works for the biodiversity survey of nematodes that are culturable, as well as other poorly studied microscopic organisms. Even non-culturable organisms can be studied using “reverse taxonomy” because the host identity and association serves as a handle for re-isolation attempts to recover the biology and morphology of the target nematode MOTU.

The insect-associated nematodes pose challenges for the application of the MOTU and morphological voucher-based (reverse taxonomy) approach because of a relatively low chance of culturability (14 cultures/51MOTUs  = 27.5% in this study). However, their high phylogenetic divergence and potential importance in natural ecosystems need further elucidation. A hierarchical approach (associative MOTU foray or transect survey and re-isolation with reverse taxonomy) has the potential to effectively expand the sequence database and associated taxonomy and biology because the molecular information is clearly tied to substrates and hosts. This approach also works to synergize modern and traditional taxonomic approaches by allowing the science to pull itself up by its own proverbial bootstraps.

The Phylum Nematoda is one of the most speciose phyla in the animal kingdom, e.g. >1 million species just from deep sea sediments [40], yet only about 25000–30000 species have been taxonomically described. There should be exceptional undiscovered functional group diversity in the world, and time consuming species-level alpha taxonomy is a major limiting factor in documenting this diversity. To accelerate the accumulation of biological and taxonomic information that is applicable to functional diversity surveys, DNA barcodes are critical [41]. Further, discovering and characterization of new lineage (functional group) with DNA barcode prior to formal description or identification, e.g. Tylenchid parasite and Rhabd1in this study, could help our understanding of diversity.

## Materials and Methods

### Overview

We collected and dissected various species of termites from several different localities in the American tropics and subtropics to obtain nematodes directly from the termite body, and sequenced a 600 bps fragment (barcode) of SSU. The barcode sequences were analyzed phylogenetically and separated into clades, which were tentatively regarded as “generic-level” resolving taxa. Then, according to the first survey, we re-sampled the termites and dissected them onto water agar plates and kept them at room temperature for several weeks to establish nematode cultures. The cultured nematodes, which were identified morphologically, and amplified and sequenced for their MOTUs, were compared back to our original MOTU “generic-level” survey to evaluate the efficiency of these two different methods.

### MOTU Surveys

The first surveys were conducted at 34 localities in three different countries. One site was in South Florida, USA, one in Costa Rica, and 32 in Panama, and the details of the locations are shown in Table S1.

The termites were collected from various environmental conditions, e.g. dead wood, under rocks, soil and hollow of living trees in focal sampling spots (La Selva, Costa Rica) [6] or along the trail (other localities). Because many species of soil-feeding termites are vulnerable to starvation and drying, the collected termites were stored in a 50 ml plastic capped centrifuge tube until dissection, and dissected within 24 hours after sampling. Twenty individual workers (or less, when numbers were not sufficient) were arbitrarily chosen from each colony, casually washed to remove the soil or frass, and dissected in a water drop under a stereomicroscope. During each dissection, the termite head capsule was cut open along with the body cavity and digestive tract to examine for endoparasitic species, and allowed to settle for a few minutes to enable phoretic nematodes to escape. Nematodes obtained from dissected termites were observed under a light microscope and individually picked and stored in nematode digestion buffer [42], [43], or if more than 10 nematodes were obtained, they were hand-picked and transferred to TSB agar plates for culturing attempts. The nematodes stored in the buffer were brought back to the laboratory, digested and heat-treated at 55°C for one hour of digestion followed by 95°C for 10 minutes to denature the proteinase K enzyme. The digested nematode served as a template for PCR amplification and MOTU sequencing analysis using the methods previously described in detail, i.e., ca. 600 bps of SSU with a primer set 18S 965 (positions 879–901: 5′-GGCGATCAGATACCGCCCTAGTT-3′) and 18S 1537R (positions 1567–1547: 5′-TACAAAGGGCAGGGACGTAAT-3′) and sequenced the amplified DNA fragment using a BigDye® Terminator v.3.1 Cycle Sequencing Kit following the manufacturer’s manual [6].

### Culturing Surveys

The second surveys were conducted in two localities, i.e. La Selva, Costa Rica and Barro Colorado Island, Panama. For the culturing surveys, all termite colonies were collected along the trail, otherwise collection methods and storage conditions were the same as the MOTU surveys. Twenty individual workers were arbitrarily chosen from each colony, casually rinsed and squashed on a 2% water agarose plate. The plates were kept at room temperature and observed daily to examine for nematode propagation. Propagating nematodes were observed under a light microscope to determine feeding habit and transferred to an appropriate media, i.e. TSB agar for bacterial feeders and a fungal lawn of *Monilinia fructicola* on GPDA for fungal feeders to establish laboratory cultures. The successfully cultured nematodes were morphologically observed under a light microscope, identified at genus or species level, and sequenced for its MOTU barcode as above [6].

### Molecular Phylogenetic Analysis

All MOTU sequences obtained here were submitted to the GenBank database and compared with other sequences deposited there to identify the closest matching nematode taxonomic and/or phylogenetic groups for each MOTU.

The molecular phylogenetic analysis was conducted using all obtained MOTUs and SSU sequences used in the previous studies [21], [22], [38], [44], [45] to construct a phylogenetic tree. The sequences compared were selected based upon the result of a GenBank homology search. The sequences were aligned using the MAFFT program [46] and the model of base substitution was evaluated using MODELTEST version 3.7 [47]. The Akaike-supported model, the log likelihood (lnL), the Akaike information criterion (AIC), the proportion of invariable sites and the gamma distribution shape parameters and substitution rates were used in phylogenetic analyses. Bayesian analysis was performed to confirm the tree topology using MrBayes 3.1.2 [48] running the chain for 1,000,000 generations and setting the ‘burn in’ at 1,000. We used MCMC (Markov Chain Monte Carlo) methods within a Bayesian framework to estimate the posterior probabilities of the phylogenetic trees [49] using the 50% majority-rule. The taxonomic groups were labeled according to the phylogenetic position (Fig. 1) and morphological identification/confirmation of the successful cultures.

## Supporting Information

Table S1 **Geographical and host/carrier information of nematodes. (EXL)**
(XLS)

Table S2 **Nematode species isolated from each termite species. (EXL)**
(XLS)

Table S3 **Accession numbers for nematode barcodes. (EXL)**
(XLS)
